# The Role of Temporomandibular Joint Arthroscopy for Diagnosis and Surgical Management of Synovial Chondromatosis

**DOI:** 10.3390/diagnostics13172837

**Published:** 2023-09-01

**Authors:** Salvatore Sembronio, Luca Raccampo, Alessandro Tel, Michele Di Cosola, Stefania Troise, Giovanni Dell’Aversana Orabona, Massimo Robiony

**Affiliations:** 1 Maxillofacial Surgery Department, Academic Hospital of Udine, Department of Medicine, University of Udine, Piazzale S. Maria della Misericordia 1, 33100 Udine, Italy; 2 Department of Clinical and Experimental Medicine, University of Foggia, 71122 Foggia, Italy; 3 Maxillofacial Unit, Department of Neuroscience, Reproductive Sciences and Dentistry, University of Naples Federico II, 80131 Naples, Italy

**Keywords:** arthroscopy, temporomandibular surgery, TMJ, synovial chondromatosis

## Abstract

Objective: We report the experience of our maxillo-facial surgery unit into the diagnostic and the therapeutic role of arthroscopy of temporomandibular joint (TMJ) synovial chondromatosis (SC). Materials and Methods: A series of sixteen patients with an imaging, arthroscopical, and histological diagnosis of SC treated with arthroscopy was selected. The surgeries were conducted in the Department of Maxillo-facial surgery, Academic hospital of Udine, from January 2016 to December 2022. Medical history, clinical examination, imaging, arthroscopical, and histological characteristics were recorded and then reviewed and discussed. Results: Clinical improvement, both in pain and in maximum incisal opening (MIO), were noticed in whole patients. Histologically, according to Milgram’s classification, the sample was fairly homogeneous. Arthroscopic treatment was successful in 87.5% of the patients. Only two cases of SC relapse were registered and were then submitted to open surgery to perform a total sinovectomy. The data collected were used to develop an SC classification proposal based on clinical, radiological (magnetic resonance imaging), arthroscopical, and histopathological characteristics. Conclusions: TMJ arthroscopy must be considered the first line of treatment for SC, leaving open surgery to relapses cases and those cases with extraarticular extension. A univocal classification is essential to best stage and prognostically characterise this pathology.

## 1. Introduction

Synovial chondromatosis (SC) is a rare and debilitating disorder defined by the World Health Organization as a benign nodular cartilaginous proliferation arising from the joint synovium, bursae, or tendon sheaths [1]. This abnormal growth is characterized by metaplastic changes and by the formation of nodules of highly cellular hyaline cartilage, which may become pedunculated and detach from the synovial membrane, forming loose bodies (LBs) within the joint space [2]. The first mention of SC dates back to 1558, by Ambroise Pare [3], followed in 1764 by Baron Albrecht von Haller, who reported the presence of LBs in the temporomandibular joint (TMJ) [4], before Georg Axhausen provided a more technical case description of TMJ’s SC in 1933 [5]. It typically affects the large joints, like the knee, hip, elbow, wrist, ankle, and shoulder, while it is relatively uncommon in the TMJ. When it occurs in the TMJ, it usually affects the upper articular space, which could lead to expansion of the joint space or capsule and intrajoint fluid collection [6]. However, involvement of the inferior joint compartment, and even extraarticular extension to the infratemporal space, the parotid region, or to the middle cranial fossa, has also been described [7,8]. While it can affect individuals of any age, SC is commonly diagnosed in young to middle-aged adults, with a higher prevalence in women; most of the patients are unilaterally affected without side predilection [9,10]. Its pathogenesis remains unclear, but two forms have been described. Primary SC pathogenesis is unknown, while the onset of secondary SC, which is linked to a more passive process, is associated with arthritic or mechanical conditions such as trauma, inflammatory or degenerative arthritis, and other joint diseases [11]. This secondary form is considered more common and less aggressive [12]. Clinical manifestations typically include pain, swelling, clicking, crepitation, facial asymmetry, functional impairment, deviation, and limitation on mouth opening. An accurate differential diagnosis is crucial to differentiate it from other types of temporomandibular disorders (TMDs), especially in the early stages where the signs and symptoms are similar and unspecific. Employing a combination of clinical assessment, imaging techniques such as computed tomography (CT) and magnetic resonance imaging (MRI), and histopathological examination is essential to confirm the presence of abnormal cartilage growth within the joint. Chen et al. [13] recently proposed a three-type classification based on MRI findings: first type: LBs; second type: homogenous mass; third type: mixture of both LBs and homogeneous mass. In 1977, SC was histologically classified into three phases by Milgram [14]: stage 1, or the early stage, involves metaplasia of the synovial membrane without the presence of LBs; stage 2, or the intermediate stage, demonstrates metaplasia of the synovial membrane with the presence of LB; stage 3, or the final stage, shows only LBs without synovial involvement. Stage 3 of SC can also include secondary calcification of the LBs, a condition referred to as Henderson–Jones syndrome [15]. Once diagnosed, treatment options for SC traditionally consist of complete LBs removal and synoviectomy, aiming to alleviate pain, improve joint function, and preserve the structural integrity of the joint. While traditional treatment approach for TMJ SC included open joint surgery or arthrotomy, which often results into significant tissue damage, prolonged recovery periods, and suboptimal outcomes, recent advancements in arthroscopic techniques have revolutionized the management of this condition. In 1989, McCain and de la Rua first reported and described the arthroscopic treatment of TMJ SC and assessed that open surgery indication must be limited to cases where the LBs are over 3 mm in diameter [16]. Arthroscopy provides a direct and magnified view of the TMJ, enabling thorough examination and diagnosis, but also the treatment of SC, exploiting a minimally invasive approach reducing surgical trauma, postoperative pain, and scarring. Additionally, this technique facilitates better preservation of healthy joint structures, and permits the surgeon to reach the medial aspect of the TMJ, which is reported not to be always possible with open surgery [17]. The aim of this article is to report the authors’ experience in treating SC with arthroscopy, clarifying its diagnostic and therapeutic role.

## 2. Materials and Methods

### 2.1. Patient Population/Study Design

This is a single-institution, retrospective study conducted in the Department of Maxillo-facial surgery, Academic hospital of Udine, from January 2016 to December 2022. A total of sixteen patients with an imaging, arthroscopical, and histological diagnosis of SC were selected. Age, gender, medical history, clinical signs and examination, imaging, and histological characteristics were recorded. Patients included in this study had completed a follow-up period of at least six months, otherwise they would have been excluded from the study, as well as patients with incongruous or missing clinical documentation. No other inclusion/exclusion criteria were established.

### 2.2. Medical History and Physical Examination

Complete medical history of the patients was collected, mainly focusing on systemic arthritic disease, previous trauma, or TMJ surgery. TMJ function was mainly assessed by measuring the maximum incisal opening (MIO), defined as the distance between the central incisors when the mouth is fully open, in addition to the assessment of lateral and protrusive movements. Moreover, the presence of swelling, functional impairment, clicking, crepitation, facial asymmetry, and deviation on mouth opening was evaluated. Patients were asked to assess preauricular pain using a visual analogue scale (VAS).

### 2.3. Imaging

All the patients included in the study performed a preoperatory imaging evaluation. An MRI, both T1-weighted and T2-weighted, in mouth closed, half, and fully open position, was performed by all patients. MRI appearances of TMJ SC like joint effusion, presence of LB, proliferative synovium, expanded joint capsule, fluid accumulation within the joint space, and extraarticular involvement were assessed. Those MRI findings were used by two of the authors (S.S. and L.R.) to classify those patients following the comprehensive classification proposal later presented in the discussion section (Table 1). Some patients also performed a CT scan, both in closed and open-mouth position, permitting us to evaluate the presence of calcified LBs and their possibility to shift location, as well as irregularity of joint surfaces, sclerosis, and hyperostosis of the glenoid fossa and mandibular condyle.

### 2.4. Surgical Technique

**Figure 1:**
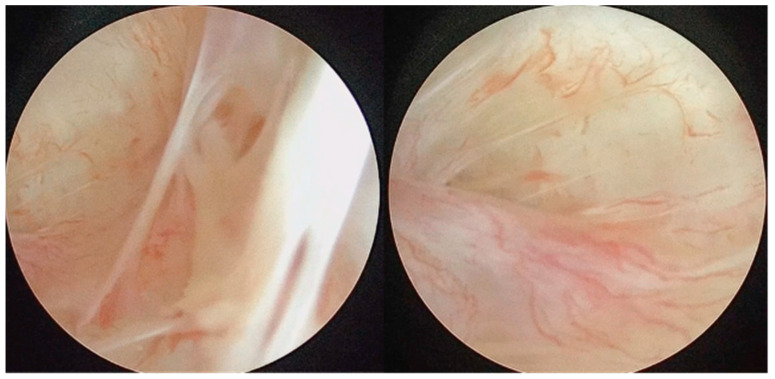
Arthroscopic view of adhesions and moderate to severe synovitis.

**Figure 2:**
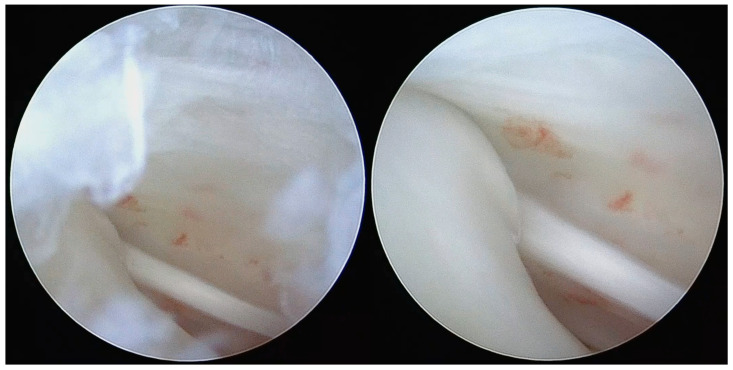
Disk perforation with condyle head exposure and exploration of inferior articular compartment.

**Figure 3:**
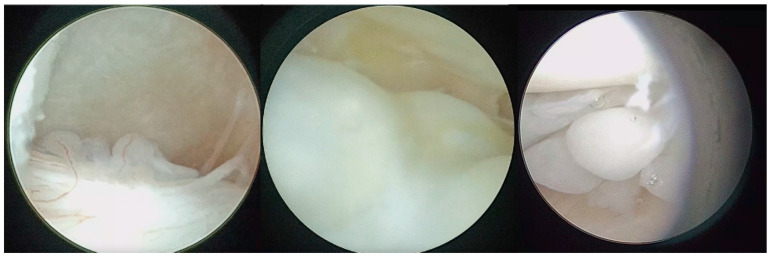
Polyps with vascular axial supply, subsynovial nodules, and LBs.

**Figure 4:**
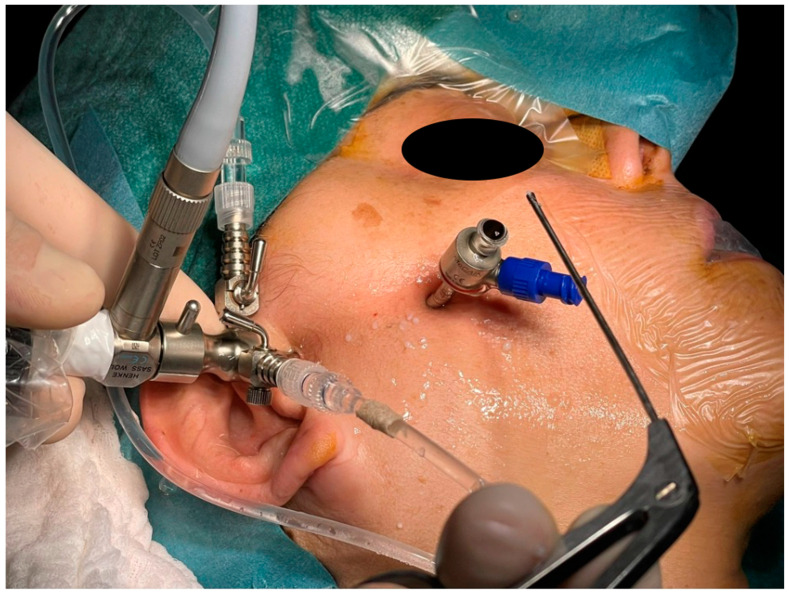
Triangulation technique and LBs washout from the second cannula, which can be seen lying on the patient’s face.

**Figure 5:**
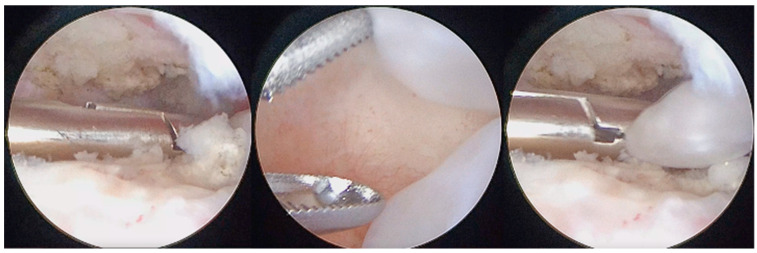
Arthroscopic removal of LBs and synovial nodules using forceps.

**Figure 6:**
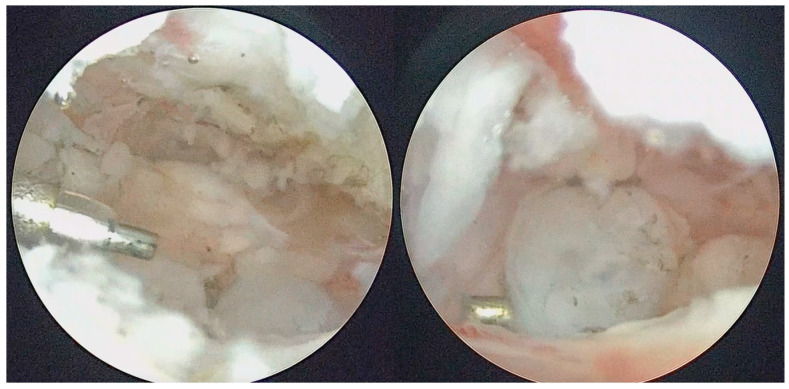
Coblation of metaplasic nodules and division of large LBs to ease their washout.

**Figure 7:**
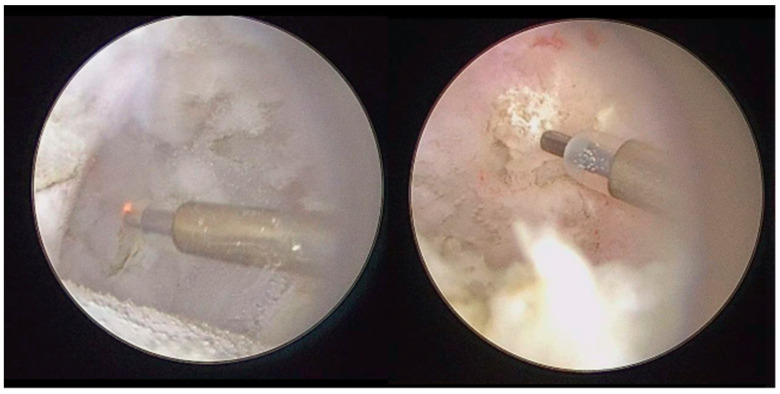
Coblation treatment of the metaplasic synovia, performing the so-called selective synoviectomy.

**Figure 8:**
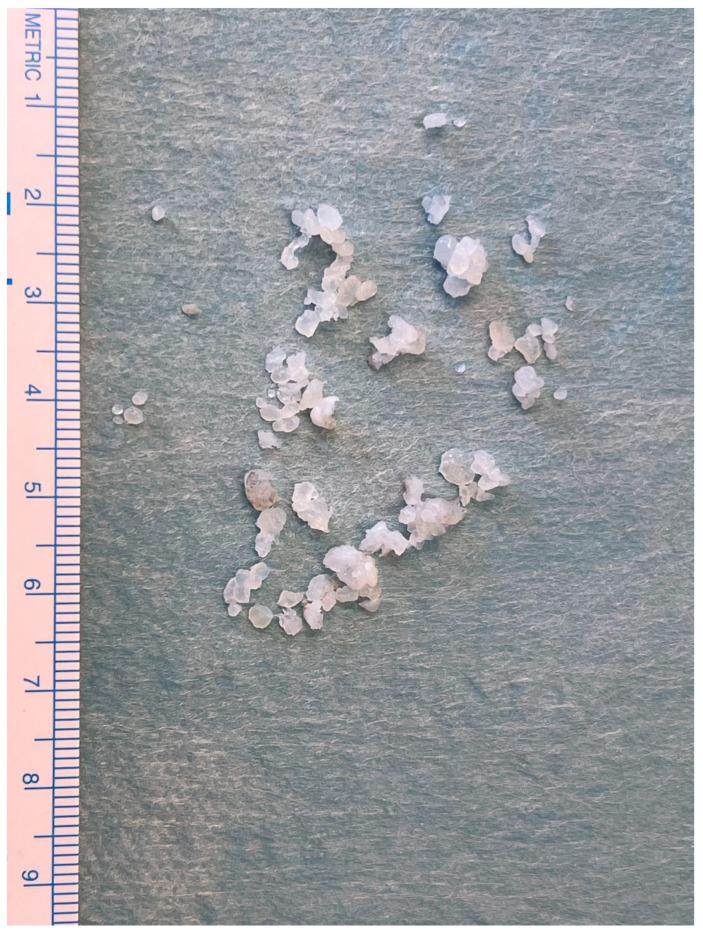
LB sample sent for histopathological analysis.

The arthroscopic treatment of TMJ chondromatosis encompasses various procedures depending on the extent of the disease and the individual patient’s needs. These include removal of LB and treatment of synovia (scarification, removing of hyperplastic synovia). All patients were treated under general anaesthesia with nasal intubation. The same surgeon performed all surgeries (S.S.). The Henke-Sass Wolf (Tuttlingen, DE) arthroscopic system (1.9 mm, 0°) was used. TMJ was identified by palpation by opening and closing the patient’s mouth. A 19 G needle was introduced in the upper compartment and saline was injected enlarging the upper joint space through a pumping technique. A small incision on the injection point was performed with a No. 11 scalpel blade. At this point, the needle was removed and the trocar with the arthroscopic sheath was inserted into the posterior recess of the upper joint space. The trocar was then removed, and the arthroscope was inserted into the arthroscopic sheath, providing a clear view of the TMJ upper compartment and of the signs of SC such as osseus contours, hyperplasia of the synovia, subsynovial nodules, nodules, LBs, synovial polyp, synovitis, chondromalacia, perforation of the disk, and adhesions (Figure 1, Figure 2 and Figure 3). This first port acted also as irrigation port, and saline was used to continuously wash out the joint space, removing any debris or remaining LBs. Using a triangulation technique, a second cannula of 2.0 mm was introduced in the anterior recess of the superior joint space, and it was used for instrument passage, drainage, and LBs evacuation (Figure 4). However, changing to a larger cannula system, like a 3.0 mm system, which may provide adequate clearance for removal of large LB, was also performed. Forceps were used to remove the LBs larger than the cannula diameter (Figure 5). LBs were also fragmentated using a cold ablation (coblation) radiofrequency device (COBLATOR^TM^ II Surgery System, Smith & Nephew, UK) (Figure 6 and Figure 7). Coblation is a process that uses a radiofrequency electrical energy passing through saline solution, producing plasma that can be applied precisely to tissues to break molecular bonds within cells. This device was also used to remove the hyperplastic synovia and perform a selective synovectomy of the metaplasic areas. The nodules attached to the subsynovial connective tissue were also precisely coblated. Moreover, coblation provides the possibility to split large LBs (>3 mm) in order to ease their washout or removal. Specimens from the affected synovia and LBs were harvested and sent to the Pathology Department to provide a definitive diagnosis (Figure 8). Manual manipulation of the mandible was performed during the approaches and through the whole surgery to reach all joint zones. At the end of the procedure, an intrarticular injection of 1 cc of hyaluronic acid was performed to ease articular mobilization and for anti-inflammatory purpose, then the arthroscopic sheath and the cannula were removed. Therefore, incisions were closed with sutures.

### 2.5. Histological Examination

The LBs and the affected synovia arthroscopically harvested specimens were sent to the Pathology Department of our hospital for histopathological examination. A histopathological diagnosis of SC was made for all patients. Milgram classification (Table 2) was used to stage all the patients.

### 2.6. Follow-Up and Outcome Evaluation

After the arthroscopic procedure, the patients were monitored in a recovery area until they were awake and stable. Pain medications and anti-inflammatory drugs were prescribed to manage postoperative pain. The patients were discharged 1 day after the surgery and follow-up clinical evaluations were scheduled 1 week, 1 month, 3 months, 6 months, and 12 months after surgery. If no problems were detected, the patients were then scheduled for an annual follow-up visit. During the first six months after surgery, all patients underwent articular physiotherapy with mandibular manipulation and joint mobilization. Clinical signs were assessed and recorded. Specifically, two parameters, such as the MIO and the VAS, were used to clinically assess patient outcome. A follow-up MRI was performed on all patients six months after surgery.

## 3. Results

**Figure 9:**
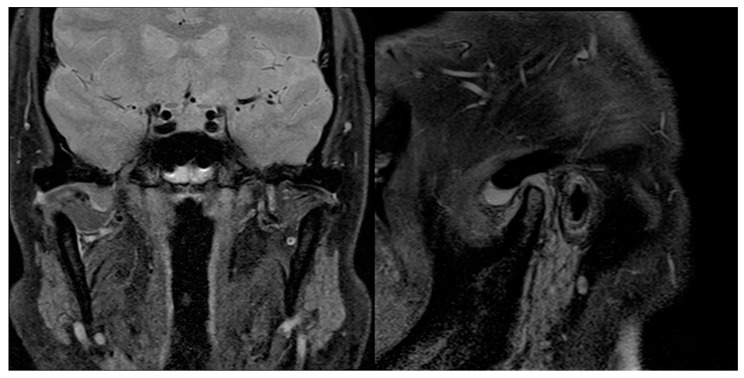
Preoperative sagittal and coronal MRI scans showing, on the right, TMJ joint effusion and LBs, but no bony involvement.

**Figure 10:**
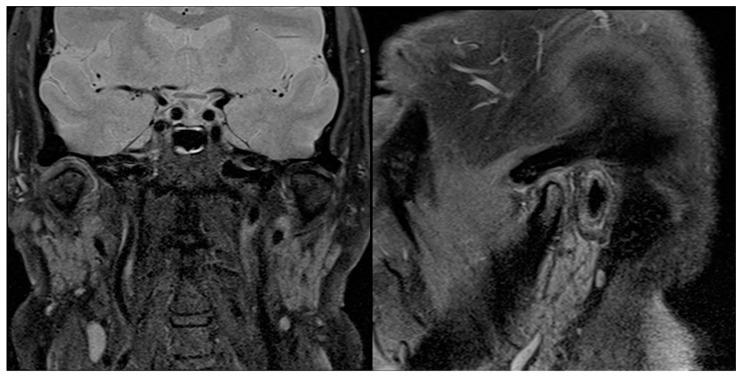
The 6-month postoperative sagittal and coronal MRI scans showing no signs of relapse on the right TMJ.

**Figure 11:**
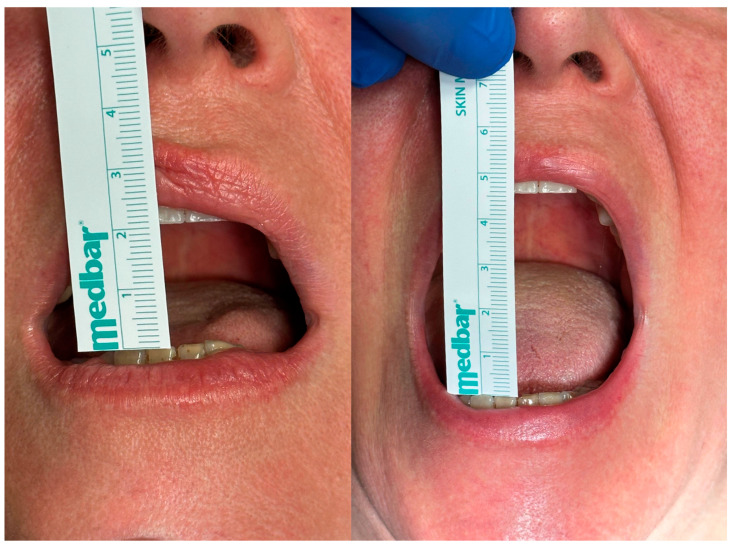
Important visible MIO improvement at 6-month clinical follow-up examination.

Between January 2016 and December 2022, 16 patients with clinical and radiological suspicion of SC underwent diagnostic and operative TMJ arthroscopy. Preoperative and postoperative clinical and imaging data (Figure 9) were recorded and are shown in Table 3. Physical examination data at the 6-month follow-up evaluation were analysed to better match the imaging data collected from the 6-month postoperative MRI (Figure 10). Patient age ranged from 24 to 72 years, with a mean of 50.9 years. A sex predominance was observed, with 13 female (81.2%) and just 3 male patients (18.8%). All case reported were monoarticular, with a small prevalence in affected joint of the left side (62.5%) compared to the right side (37.5%). Physical examination records showed a preoperative MIO ranging from 20 to 38 mm with a median of 29.6 mm, while the postoperative MIO ranged from 25 to 48 with a median of 37.9 mm, showing an 8 mm MIO improvement after surgery (Figure 11). Pain assessed with VAS showed a significant improvement of almost 5 units, with the preoperative values ranging from 6 to 8 with a median of 7.1 and the VAS assessed six months after surgery ranging from 0 to 7 with a median of 2.6. All 16 patients were submitted to an MRI before the surgery, while a CT scan was performed in just five patients. The diagnosis of SC was confirmed by histopathological examination and classified following Milgram classification: six patients (37.5%) were classified as a stage 1, as many as the ones classified as stage 2, while four patients (25%) were classified as stage 3. There were no extracapsular soft-tissue involvements that were proven by pathology and MRI in all subjects. It was not possible from the data collected to determine with certainty which form of SC, primary or secondary, the patients were suffering from. The follow-up period ranged from 8 to 71, months with a mean value of 30.6 months. Only 2 of the 16 patients (12.5%) showed a recurrence of SC, and in both cases, this was noticed at the 6-month follow-up evaluation. These two patients were then submitted to open surgery within 6 months of the relapse diagnosis. One of these two patients needed a second arthrotomy 18 months after the first one, because another relapse of SC was noticed at the follow-up MRI.

## 4. Discussion

SC was considered a rare condition affecting the TMJ in the past, while recently, reports on the disease have increased. Overviewing the literature, there were mainly reported cases of advanced stage of SC of the TMJ according to Milgram’s classification [14]. This could be attributed to the clinical and radiological similarity of SC to other TMDs, which could lead to initial misdiagnosis. We instead reported just three cases of Milgram’s stage 3. However, a trend of progressive reduction in the time between occurrence of the first symptoms and provisional diagnosis of SC was recently addressed [18]. A role in this tendency could be represented by the MRI evolution, because this imaging technique is the only one which permits the visualization of the radiological signs of Milgram stage 1. In fact, the absence of LBs does not exclude SC, as the radiographic demonstration of LBs depends on the extent of calcification, and MRI allows the identification of cases not identified on CT, as it can visualise LBs in the early stages [9]. The calcified LBs are often seen in MRI as low and iso-intensity signal nodules of both small round and punctuate forms [18]. Moreover MRI, with its superior contrast resolution, is useful for showing the extension and boundaries of the lesion, assessing internal derangement of the TMJ, and confirming synovial origin of the lesion [19]. MRI SC features mainly include joint effusion, which is best noticed on T2-weighted sequences, LB within the joint space, proliferative synovium, expanded joint capsule, and anterior displacement of the mandibular condyle [9,13,18,19]. Another advantage of MRI over CT is the early detection of extraarticular extension. The MRI is not only useful for the diagnosis of SC, but it also represents the gold standard for follow-up after surgery, which we used to highlight signs of relapse in 2 of the 16 patients. Moreover, since the treatment is surgical, a thorough radiological evaluation is essential to choose the best type of surgery. SC generally occurs in the superior joint space, which could be due to the fact that the superior compartment is larger than the inferior one, and therefore its capacity to produce LBs is greater than that of the inferior compartment, as some have suggested [20,21]. This predilection also makes arthroscopic management of SC feasible as a diagnostic, but especially operative, technique [12]. In view of the various elements that characterise the pathology, and the lack of n univocal classification, the authors propose a classification that encompasses the clinical, radiological MRI, arthroscopical, and histopathological features of SC, the latter expressed by Milgram’s classification (Table 1). This follows the principles of the classification of TMDs according to Wilkes [22]. This classification allows and simplifies a complete staging by also providing a prognostic point of view of the disease but obviously needs to be validated. The aim of this proposal is to stimulate experts of the field to assess an unambiguous classification that can best describe the pathology, considering the arthroscopic point of view as well as the histological and radiological ones. The accepted treatment of SC consists of the complete removal of the LBs and synovectomy of the affected synovia. This was historically achieved by open arthrotomy, while in recent years, arthroscopy has been reported to be almost equally effective in selected patients, moving away from its sole diagnostic role [23]. In the past, it was generally stated that open surgery was required if the loose body is over 3 mm in diameter. This is no longer true, thanks to the advancements into the development of arthroscopic instruments which permit us to fragment the LBs [24]. Arthroscopy can also show areas of metaplasic changes of the synovia, which can be coblated with radiofrequency devices. It also permits synovial biopsies to confirm the pathology. Cai et al. [25] previously reported their experience in the arthroscopic treatment of 33 patients affected by SC and broadened the indications based on MRI diagnosis. We have previously stated the importance of arthroscopy even as a complementary approach to open surgery [26], due also to the fact that this technique best permits us to reach the medial aspect of the joint. In aggressive cases with extraarticular extension, or when an involvement of the lower compartment is highlighted, open surgery remains the therapeutic modality of choice. It is usually performed by a preauricular approach, with removal of loose bodies and complete synovectomy. The trend should be that the stage of the disease should guide the choice of the surgical approach. Recurrence is rare in SC but is more frequent in primary SC and in cases of extraarticular extension [27]. Arthroscopy showed a slightly higher rate of relapse than open arthrotomy [28]. The idea is based on the fact that remnants of the synovium may become a source of recurrence, while others state that the condition has a self-limiting character, so that total synovectomy may be unnecessary [29]. As stated before, we reported a recurrence rate of 12.5%, which occurred in Milgram stage 1 and stage 2 patients; thus, in whom the stage indicates a high level of metaplastic proliferation. Those relapses were detected within 6 months from the arthroscopy, making this period adequate for the assessment of recurrence. One of the patients showed a relapse even after the arthrotomy was performed and needed a second open surgery. The patient was classified as a Milgram stage 2. This could indicate that in more proliferative stages even an open arthrotomy may not be sufficient to perform a complete synovectomy. Another possibility is that in the first arthrotomy some remnants of the affected synovia were left in the TMJ. Arthroscopy showed a significant impact on clinical improvement. Both pain, assessed with VAS, and MIO were greatly improved. This corroborates the hypothesis that arthroscopy may represent a more than feasible option to treat this pathology.

## 5. Conclusions

Understanding this disorder is crucial for clinicians as it allows for early detection, effective management, and improved quality of life for those affected by this condition. Certainly, arthroscopy has a central role in diagnosis of TMJ SC. Most of all, arthroscopy represents a valid option for the treatment of SC. It must be considered the first line of treatment for SC, leaving open surgery to relapses cases and those cases with extraarticular extension. Considering other diagnostic clinical values and increasing the research population could be essential to assess and certify the role of arthroscopy in TMJ SC. Moreover, also in view of the classification proposed here, it is essential to best classify the pathology. Collaboration between clinicians and researchers is crucial in developing standardized protocols and guidelines to ensure the widespread adoption of arthroscopic treatment as the gold standard in managing TMJ SC.

## Figures and Tables

**Table 1:** The comprehensive clinical, radiological, arthroscopical, and histopathological classification proposal presented here. It includes three stages of increasing severity, of which elements could overlap, but most of the matching elements establish the stage. It aims to have a diagnostic but also a prognostic role.

Stage	Clinical Evaluation	MRI Appearance	Arthroscopic Inspection	Histopathological Findings (Milgram)
1	Painless or with occasional pain; maybe present some articular sound; MMO in normal range.	Effusion with no LB; proliferative synovia; no bony alterations.	Normal osseus contours; synovitis; hyperplasia of the synovia; no LB or nodules.	Involves metaplasia of the synovial membrane without the presence of LBs.
2	Patient with frequent pain; joint tenderness; articular sound; reduction of the MMO (35–25 mm).	Effusion with LB; proliferative synovia; initial bony alterations; fluid accumulation within the joint space.	Normal osseus contours; hyperplasia of the synovia with subsynovial nodules; nodules about to detach and LBs; synovial polyp; synovitis.	Demonstrates metaplasia of the synovial membrane with the presence of LBs.
3	Patient with chronic pain sometimes of various entities, headache, joint tenderness; reduction of the MMO (>25 mm).	LB; proliferative synovia; bony alterations and extraarticular involvement; fluid accumulation within the joint space.	Abnormal bone contours and degenerative osseus changes; no articular joint capsule hyperplasia but just LBs; synovial polyp; synovitis, chondromalacia; perforation of the disk; adhesions.	Shows only LBs without synovial involvement.

**Table 2:** Milgram’s histopathological classification published in 1977.

Stage	Histopathological Findings (Milgram)
1	Involves metaplasia of the synovial membrane without the presence of LBs.
2	Demonstrates metaplasia of the synovial membrane with the presence of LBs.
3	Shows only LBs without synovial involvement.

**Table 3:** Table of our study data. T1 represents a preoperative statement, while T2 represents the 6-month postoperative time.

ID	Sex	Date of Birth	Age (Years)	Surgery Year	Affected TMJ	MIO T1 (mm)	MIO T2 (mm)	VAS T1	VAS T2	MRI	CT	Milgram	Relapse	Follow-Up Period (Months)	Conversion to Open Surgery
1	F	1962	55	2017	Left	35	37	7	3	X	NO	2	NO	71	NO
2	M	1952	66	2018	Right	35	36	6	3	X	NO	1	NO	65	NO
3	F	1969	49	2018	Left	20	25	8	4	X	X	1	YES	60	YES
4	F	1970	49	2019	Left	30	43	6	1	X	NO	2	NO	47	NO
5	F	1952	67	2019	Right	38	48	6	2	X	NO	2	NO	45	NO
6	M	1992	27	2019	Left	30	41	7	1	X	X	2	NO	44	NO
7	F	1979	40	2019	Left	29	45	8	7	X	X	2	YES	43	YES
8	F	1969	52	2021	Right	28	25	8	5	X	X	1	NO	22	NO
9	F	1973	48	2021	Right	28	37	7	0	X	X	1	NO	21	NO
10	F	1954	68	2022	Left	21	45	8	0	X	NO	3	NO	13	NO
11	F	1985	37	2022	Left	25	34	8	3	X	NO	1	NO	12	NO
12	F	1950	72	2022	Right	35	44	7	2	X	NO	3	NO	12	NO
13	F	1998	24	2022	Right	31	38	6	2	X	NO	1	NO	10	NO
14	M	1964	59	2023	Left	37	40	8	1	X	NO	3	NO	9	NO
15	F	1978	45	2023	Left	27	35	7	4	X	NO	2	NO	8	NO
16	F	1966	57	2023	Left	25	34	7	3	X	NO	3	NO	8	NO

Identification (ID); sex: female (F), male (M); maximum incisal opening (MIO); T1 represents a preoperative statement; T2 indicates the 6-month postoperative time; visual analogue scale (VAS); magnetic resonance imaging (MRI); computed tomography (CT).

## Data Availability

Not applicable.
